# Exploring health information system resilience during COVID-19 pandemic: case studies from Norway, Sri Lanka & Rwanda

**DOI:** 10.1186/s12913-023-10232-0

**Published:** 2023-12-18

**Authors:** Pamod Madusanka Amarakoon, Ragnhild Bassøe Gundersen, Andrew Muhire, Vetle Alvenes Utvik, Jørn Braa

**Affiliations:** University of Oslo, Oslo, Norway

**Keywords:** Resilience, Sri Lanka, Norway, Rwanda, Information systems, Health system resilience

## Abstract

The study aims at exploring health system resilience by defining the scope on health information systems, one of the six building blocks of the health system. The empirical evidence is derived using qualitative data collection and analysis in the context of Norway, Sri Lanka and Rwanda during the COVID-19 pandemic. The case studies elicit bounce back and bounce forward properties as well as the agility as major attributes of resilience present across the countries. Existing local capacity, networking and collaborations, flexible digital platforms and enabling antecedent conditions are identified as socio-technical determinants of information system resilience based on the case studies across the countries.

## Introduction

Drawing on three interpretive longitudinal case studies from Sri Lanka, Rwanda and Norway, this paper describes similarities and differences in the countries´ digitalization processes, in relation to resilience and rapid response. Rapid implementation can be defined as in corporating speed and efficiency, while having the ability to adapt methods and trial design to suit the needs of complex studies [1]. We show how the three countries worked to establish health information systems (HIS) to handle the COVID-19 pandemic. HIS is one of six building blocks World Health Organization (WHO) constitutes as the health system of a country [2]. Destabilization of any of the building blocks could lead to jeopardizing the health system. We are especially motivated by the impact of health information in ensuring the resilience of the health system.

A health system of a country is a major source of provision of welfare to its population. ‘Ensuring healthy lives and promoting well-being for all at all ages’ is the third goal of the United Nations sustainable development goals, which highlight the prominence provided to health systems across all countries [3]. Numerous catastrophic occurrences or shocks, like the financial crisis or the Ebola outbreak, have recently had an impact on health systems all over the world. Some of the health systems seemed to have been more capable of handling these events and/or better able to anticipate them than others. In other words, some had stronger shock resistance and/or shock responses.

Most recently, the emergence and quick spread of COVID-19, has put the world's health systems to the test, driven the world economy in jeopardy, and as a result, has caused human costs that far outweigh the disease's actual effects. Global health systems and economics have been severely damaged by COVID-19, particularly in developing nations. With limited resources, developing nations have been striving to treat the pre-existing disease burden, which was made considerably more difficult during COVID-19. The high cost of treatments, market failures in pluralistic health systems, high out-of-pocket costs, the extra burden of noncommunicable illnesses, missing business opportunities, and social effects including unemployment and poverty are only a few of the economic effects of COVID-19 in those nations [4].

While developed countries generally have efficient health systems, they were overwhelmed and struggled to provide sufficient care to individuals during the first phase of the COVID-19 pandemic. However, an almost abundance of resources assisted the developed countries to combat the pandemic in a robust way [5]. Thus, COVID-19 poses an intriguing question on assurance of stability of health systems in affluent nations and their ability to bounce back in unfavourable conditions.

Capacity to maintain high quality healthcare services by adapting to changes and challenges at different levels can be described as healthcare resilience [6]. Thus, adaptation and bouncing-back are inherent properties of a system's resilience. Due to the variability of responsiveness and of country health systems´ resilience during adverse circumstances, it would be interesting to explore the determinants of rapid response and resilience at country level.

‘Resilience’ is a trending area in the information systems (IS) domain in recent times. While IS primarily deals with technology, an interesting point to note when referring to ‘implementation of information systems’ is that factors contributing to adaptation and resilience need to be of socio-technical nature. In addition to the core technical dimensions, the researcher needs to focus on organizational and broader social determinants of beneficiaries interacting with the HIS. However, there is a vacuum in literature that explores the socio-technical determinants of resilience of IS. The broader objectives of this paper is to identify key attributes of resilience of information systems and to understand socio-technical determinants involved in the process of building resilience in a country context. The empirical evidence for the study builds on interpretive case studies of the digital and informational responses to the COVID-19 pandemic in Norway, Sri Lanka, and Rwanda, which captures examples of the unfolding process of building resilience and launching a rapid response in shifting circumstances during a pandemic.

Traditionally, in the context of developing countries, the components and workflows in HIS follows a path dependent process in data collection to use. Therefore, when the process is disrupted at a certain point it requires bouncing back to attain the establishment of information flow [7]. The capacity of HIS to break path dependency and produce multi-scalar effects, reflecting new and systemic thresholds, would also be intriguing to investigate since it suggests a potential for transformability [8].

The rest of the paper is structured as follows. First, we present the existing literature on resilience. Next, in the methods section, we describe the three research approaches from Sri Lanka, Norway and Rwanda. Then, we elaborate the 3 case studies from Sri Lanka, Rwanda and Norway based on the empirical data collection, in the case studies section. Before concluding by summarizing the salient takeaways from the three case studies and potential future research, we discuss attributes for building resilience identified through the case studies, as well as the determinants which lead to establishing resilience of health information in a country.

## Theoretical framing

### Resilience

Varied disciplines, including psychology, ecology, management, disaster management, engineering, sociology, and regional development, have different definitions for resilience. Ecology pioneered the concept of resilience to describe how quickly equilibrium may be restored and how resilient complex natural systems are to change [9]. A "resilience thinking" process framework was conceived by Folke et al. [8],incorporating the concepts of resilience, adaptation, and transformability in complex social-ecological systems (SES). Resilience in such systems refers to the SES's ability to evolve and adapt continuously while staying within crucial thresholds.

From an individual’s perspective resilience has been defined as “...the positive capacity of people to cope with stress and catastrophe” [10]. “Resilient people and communities are more inclined to see problems as opportunities for growth” (Ibid.). Additionally, resilient people feel less linkage to a particular place [11], which makes it easier for them to transition into a different role in a new environment.

Since the 2014 Ebola outbreaks in West Africa, discussions of resilience have dominated health systems research, and they have become more prominent with the COVID-19 pandemic [12]. The WHO Bulletin noted that "weak health systems cannot be resilient" and that "a strong health system decreases a country's vulnerability to risks and ensures a high level of preparedness to mitigate the impact of crisis," as Ebola devastated the inadequate systems in Liberia, Sierra Leone, and Guinea [13]. Effective "information systems" and robust epidemiological surveillance were cited in a WHO brief as essential resilience-building measures [14].

Sochas et al. [15] estimated that the decreased utilization and provision of routine health services for reproductive, maternal, and neonatal health, led to a similar number of deaths as those directly from Ebola in their study of "indirect crisis-related deaths in the context of a low-resilience health system" in Sierra Leone. In their analysis, they came to the conclusion that "indirect mortality effects of a crisis in a health system lacking resilience may be as important as the direct mortality effects of the crisis itself." The Lancet defines resilient health systems as having the ability to prepare for and respond to disaster, maintain routine functioning when a crisis hits, and reconfigure itself as a result of the lessons learned from the Ebola outbreak [16].

Similar to this, Blanchet et al. [17]expanded on ecology and complexity science to define resilience as the ability of a health system to absorb, adapt, and transform when subjected to stress while maintaining control over its structure and functions. The degree of structural change and crisis intensity are considered as influencing the levels of absorptive, adaptive, and transformative capacities. The process of structural change and transformation becomes more radical the harsher the crisis, necessitating the health system to adjust organizationally in order to provide the same level of healthcare services with different or less resources. A health system's transformative potential is its power to change its operations and organizational layout in response to a changing environment by creating new knowledge systems [17].

Resilience is mostly explored in information systems (IS) research as an extension of engineering studies, supply chains, infrastructure, and risk management. Heeks and Ospina [18] note the inadequate response to the question "resilience of what" and criticize the lack of development in the research of resilience in IS. One of the six fundamental components of health systems, along with the health workforce, finances, health services, medicine, finances, and governance, is the HIS [19]. With the use of this paradigm, we are urged to abandon the distinction between target and object systems and consider how closely the health system is integrated with digital resilience. The health system, such as the technologies chosen, impacts the physicality of the digital as well (eg: expanding the scale of data reporting through digital means).

Resilience traits are described by several scholars as robustness, redundancy, resourcefulness, and swiftness [20]. The analytical acuity of the notion is jeopardized by an attribute-focused approach's tendency to be overly general, and everything ends up being redundantly labelled as "resilience." In addition to the problems mentioned by Bruneau et al. [20], Klein et al. [21] offer adaptive capacity as the overarching idea for resilience. They further expand it to encompass reduced vulnerability, flexibility, adaptability, agility, and self-organization [18, 22], During the COVID-19 pandemic, Floetgen et al. [23] place special emphasis on the materiality of digital in mobility platforms and how socio-technical elements and digital ecosystems are merged and used to build resilience. By combining forward-looking actions with platform and ecosystem principles, resilience may be built inexpensively and transformatively [23].

Drawing on the extant literature on resilience, we describe an information system as resilient when it is able to evolve and adapt continuously while staying within crucial thresholds [8], implying that it is persistent, adaptive and transformative.

## Methods

In this section, we illustrate the research design, data collection method and the analysis approach.

This paper employs an interpretive case study technique [24, 25] since it enables us to examine the dynamics and context of the phenomenon of the rapid creation of pandemic-resistant digital technologies in three different nations. In contrast to more realist views, which believe in an objective, external reality, we take an interpretivist viewpoint, believing that reality is a social creation by human agents [26]. An interpretive case study method [24, 27, 28] was used to investigate the actions and perspectives of human stakeholders involved in the introduction of digital technology in the health sector during a pandemic. Such an approach, according to Darke et al. [26], is useful in developing and understudied study fields, similar to the ones examined in this paper. Our approach was an empirical investigation of a contemporary event in a real-life environment, in accordance with [29] Yin's [30] description definition of a case study, as a detailed examination of one example, enabling the researcher to test different views in relation to phenomena as they evolve, by closing in on an authentic situation. The data collection techniques used in this instance, according to Yin, were varied and included socio-technical methodologies [24, 31].

### Study setting

The main criteria for selecting the study context are the feasibility of access for conducting research during the pandemic. We opted for 3 countries that were utilizing the free and open-source health management information platform, DHIS2 [32]. The platform is currently being implemented in over 80 countries globally [33]. We selected Sri Lanka and Rwanda from LMIC context and Norway from the context of high-income countries. The authors had significant access to the implementation of digital information systems of the three countries and also had long-term engagement with the health systems of the three countries. For the scope of this study, public health digital implementations of the three countries were considered. The engagements for the research purpose were primarily with the ministries of health, academic institutions, partners supporting the implementation of the information systems and the end users at health facility level of public health sector.

In Norway, there were two suppliers of contact tracing systems that shared a user base consisting of almost all the municipalities, Fiks contact tracing being one of them, and Remin the other. Additionally, three municipalities developed their own contact tracing systems. For this research, however, we chose to study the development of Fiks contact tracing system, which is based on dhis2, the same platform used by Sri Lanka and Rwanda. By comparing the implementation of the same HIS in three different countries, we were able to describe and discuss the resilience of a HIS in three different contexts.

### Data collection methods

Data collection methods devised in each country are slightly different to each other depending on the context. The primary qualitative data collection methods that were devised in the study includes interviews, observations, analysis of meeting notes and documents. The data collection was performed to achieve two overarching objectives. Firstly, to capture how events transpired during the pandemic, and secondly, to reconstruct the historical events around the establishment of background and infrastructure in the health sector. Hence, none of the three studies followed a top-down method where we first developed a conceptual scheme and then conducted a fieldwork to confirm it. Conversely, the theoretical concepts described above, are a result of an iterative process of data collection and analysis.

In all three cases, the choice of selection of interviewees is a result of a combination of convenience and snowballing. However, the interviewees had to satisfy some criteria: knowledge and experience with surveillance and vaccination, involvement in the digitalization of COVID-19 related data management at some level. We did not require that the interviewees had to have a specific number of years of experience with related work in the health sector, the experience could be a couple of months or weeks only, because we knew that in some sites individuals had been employed as contact tracers during the pandemic. There were no exclusion criteria of participants in Norway and Rwanda. However, in the context of Sri Lanka the participants who were selected were the ones who could converse in either Sinhala or English language.

Qualitative data collection methods from Sri Lanka included interviews from stakeholders at the ministry of health, academia and the multi sector organizations. The interviews were complemented by observations and meeting notes to formulate the case studies. Rwanda’s case study was formulated by interviews, meeting notes and observations. The case study in Norway included data collection through interviews with representatives from 6 municipalities involved with the digitalization of Fiks contact tracing, representatives from KS, as well as the HISP UiO team, and senior advisors from the Norwegian Institute of Public Health (NIPH), and observations and meetings. All interviewees have been anonymized.

On some occasions the interviewees were chosen due to easier access than others, primarily due to restrictions that were posed because of the pandemic. We estimated that it would be easier to access locations closer to us, if or when the travel restrictions were lifted. However, through interviews in one area we learned about challenges in different locations, which led us to study these areas as well; the choice of a new site “followed logically from the first and so on” [34].

Interview guides were prepared based on findings in extant literature related to information system in health sector. Each interview lasted between 30 minutes and 1.5 hours. Extensive notes were taken during the interviews. The interviews were recorded digitally and transcribed in English or local language of the three countries. The transcribed interview notes were translated to English prior to analysis. The initial interview guide prepared based on HIS literature was refined based on findings from the interviews to be used in subsequent interviews. Interviews with interviewees representing distinct voices in various organizations, followed by an analysis of what the interviewees have said, was an important way towards the point of saturation, i.e. when no new insights were “being discovered in the interviews” [34].

In addition to primary data, we have analyzed publicly available reports, minutes from meetings and research reports that have described and discussed the handling of the pandemic. Updates on public service websites, such as the ministry of health, have been useful, because that has informed us of the continuous changes in rules and restrictions.

Summary of data collection methods for each country are illustrated in the Table 1 below. The table has been primarily categorised based on the job profile of the interviewees. In addition it outlines several other characteristics of the interviewees such as the location they are currently practicing based on the availability of resources (low to high), experience in the type of job role based on number of years, their age, their background expertise and the digital literacy.

**Table 1:** Data collection methods

	**Sri Lanka**	
**Interviews**	**Total Number: 32**	
	**Profile of Interviewees**	
	Administrators at MoH (3)	Selected administrators had experience working in low and high-resource settings throughout the country. Experience of administrators ranged from 10-20 years – (medium to high). All the participants were in their forties and fifties. All of them started their careers as clinicians and specialised in medical administrators All of them had moderate digital literacy
	Medical officers of Health Informatics (6)	Interviewees had experience working in low to high-resource settings throughout the country. Experience of informaticians ranged from 3-12 years – (low to high). All the participants were in their fourth, and fifth decades of their lives. All of them started their careers as clinicians and specialised in health informatics Participants had high level of digital literacy
	National level implementers (4)	Interviewees had experience working in low to high-resource settings in several districts and national levl. Experience of implementers ranged from 3-12 years in implementing systems – (low to high). All the participants were in their fourth, and fifth decades of their lives. All of them started their careers as clinicians and specialised in health informatics with some experience in public health Participants had high level of digital literacy
	District level implementers (3)	Interviewees had experience working in low to high-resource settings in 1-3 districts. Experience of implementers ranged from 2-14 years in implementing systems – (low to high). All the participants were in their fourth, and fifth decades of their lives. All of them started their careers as clinicians and specialised in health informatics with some experience in public health Participants had high level of digital literacy
	Core training team (4)	Core team had experience working in low to high-resource settings throughout the country. Experience of core team ranged from 8-11 years in implementing systems – (medium to high) in Sri Lanka and globally. All the participants were in their fourth, and fifth decades of their lives. All of them started their careers as clinicians and specialised in health informatics with public health and administration experietise. Participants had high level of digital literacy.
	End users of the system at hospital and MOH level (10)	End users consisted of medical officers, nurses, public health midwives and data entry operators. Most of the participants had experience working in mid to high-resource settings in 1-3 districts of the country. Experience of end users ranged from 2-15 years in health sector. Some data entry operators had work experience in non-health domains. Participants were in their third, fourth, fifth and sixth decades of their lives. All of them started their careers as clinicians and specialised in health informatics with public health and administration experietise. Participants had low - moderate level of digital literacy.
	Academic staff of University of Colombo (2)	Academic staff had experience working in high-resource academic institutes but with field experience in low-high resource settings. Experience of academic 10-15 years in digital systems – (medium to high) in Sri Lanka and globally. All the participants were in the fifth decades of their lives. All of them started their careers as clinicians and specialised in health informatics with public health and medical education experietise. Participants had high level of digital literacy.
**Meeting Notes**	Planning meetings of modules of the system	
	Implementation planning meetings	
	Progress review meetings	
**Observations**	Stakeholder meetings held at MoH	
	Trainings conducted at national, district and field level	
	Engagement with information system observed at data entry/analysis	
	**Rwanda**	
**Interviews**	**Total Number: 30**	
	**Profile of Interviewees**	
	Monitoring and evaluation teams at MOH (3)	2 Public health professionals and 1 epidemiologist Interviewees had several years experience from disease surveillance and M&E activities in the Ministry of Health
	National Level programs teams (3)	2 Public health professionals and 1 physician Interviewees had experience from vaccination program (EPI) and disease surveillance program
	District Hospitals implementers (3)	1 hospital manager, 1 statistician, 1 HIS specialist Interviewees had many years experience from working in hospitals
	Digitalisation teams (4)	2 IT professionals and 1 Business analysts, 1 lab technician with very good IT skills. Interviewees had many years of experience from implementing and developing IT solutions in the health sector. The lab technician had experience from computerising the manual lab system
	Planning teams at Central level (2)	Both had background in accounting and project management. Both had many years experience as project managers
	End users of the systems at Hospitals and Health centers (8)	1 Data manager, 1 Manager of the health centre, 3 Nurses, 3 lab technicians, IT staff Interviewees were all direct users of the system at the local level and they all had several years of practice in their jobs
	Health management information systems teams (3)	1 IT professional, 1 data manager, 1 M&E staff Interviewees had many years work in implementing HIS and in training users, getting feedback from users, and in help improving data systems
	Program level meeting with Hospitals (4)	1 Hospital manager, 1 data manager, 2 lab technicians. Interviewees had several years of experience in their jobs and also related to labs and the logistics related to taking samples, analysing results and providing feedback – sending back results
**Meeting Notes**	Hospital Data Coordination meeting	
	Central lab coordination meeting with participation from other labs	
	Hospital Service level meetings	
	Trainings of end users	
	Supervision at Hospitals	
**Observations**	Health facility integrated supervision visits	
	***Norway***	
**Interviews**	**Total Number: 45**	
	**Profile of Interviewees**	
	Municipality contact tracers (9)	Diverse background (from pilots to doctors to shopkeepers to students). Experience with contact tracing ranged between none and 20+ years. Age ranged from mid-twenties to mid-sixties. Some were highly digital literate and curious, while others had little experience with the digital but experience from other areas that made them question taken-for-granted practices, influencing change. The six municipalities are resourceful, two are centrally located (close to Oslo), four are in the outskirts.
	Chief municipal physicians (6)	All are medical doctors. Experience with contact tracing and disease surveillance for 10+ years. Age ranged between 40 – 70. They were all highly digital literate, and three contributed to the digitalization process.
	Municipality project managers (15)	Diverse backgrounds (Business administration, nurse, IT). Their experience working in the municipality ranged from 1 year to 20+ years. Age ranged between 40 and 60 years. They were all highly digital literate, some with information technology education and work. experience and others with a genuine interest in the digitalization process.
	National Institute of Public Health senior advisors (7)	Diverse background (epidemiologist, nurse, medical doctor). Their experience with disease surveillance ranged between 1 and 10 years. Their age ranged between 30 and 40 years. They were highly digital literate.
	HISP Oslo implementers (3)	IT Backgrounds. 10+ years of experience with IT. Ages ranging from 30 – 40. Highly digital literate.
	KS IT architects (3)	IT background. 10+ years of experience with IT. Ages ranging from 40 – 60. Highly digital literate.
	KS project administrators (2)	Backgrounds from business and administration, and IT. 10+ years of experience. Ages ranging from 40 – 60. Medium to highly digital literate.
**Meeting Notes**	Chief municipal physician meetings	
	User forum meetings	
	Corona clinic administration meetings	
**Observations**	Corona Clinic	
**Document and report studies**	Laws and regulations concerning infectious disease surveillance	
	Minutes from municipality meetings publicly available on their websites	
	Release notes from Fiks Contact tracing	
	Measures and restrictions published by national health authorities on their websites	
	Reports on the government´s and the municipalities´ handling of the COVID-19 pandemic	

### Data analysis

Our data analysis draws on a Hermeneutic approach, trying to understand and interpret our interviewees’ thinking, as well as adopt an understanding analysis of published reports and published documents available online [35–38]. This entails that to understand the individual parts we need to have an understanding of the whole, following the principle of the hermeneutic circle [38]. The principle of the Hermeneutic circle is the first principle in Klein and Myers’ [38] seven principles for interpretive field research, which are all related to the hermeneutic approach. The principles are meant as ideas and not deemed mandatory, however, interpretive researchers are expected to discuss the principles and together decide which of the principles they will apply in their project [38]. Throughout our iterative collection and analysis of our data, we have been guided by these principles.

As the digitalization in the three countries has been a continuous change-processes throughout our research, we have collected and analyzed data in a manner to enable an understanding of our interviewees as contributing to the change-process and not a mere product of history, which concurs with the principle of contextualisation. For instance, during the authors sensed a much broader collaboration and interdisciplinarity than what is common in all three contexts, that the pandemic and needed digitalization process in the three countries forced the actors to think outside the box.

During our iterative process of data collection and analysis, we inductively identified resilience as a recurring theme. Discussing our findings in relation to the concept of resilience adheres to Klein and Myers’ [38] principle of abstraction and generalization. By iteratively drawing on the concept of resilience and collecting new data, we developed a broadened understanding of resilience. Resilience thus became valuable as a sensitizing device during the fieldwork of the three countries. Walsham [24] also states that during a longitudinal case study it is expected that theory evolves in relation to an iterative process of collection and analysis of data.

Through interviews, observations and document studies we sought multiple interpretations of the case we study, a triangulation of researchers, which is important given that the social context may influence how actors behave. Our data analysis consisted of us carefully reading and reflecting on our field notes. We used NVivo to create codes and themes, preparing our data by identifying and combining interviewees’ statements that had similar meanings. Further we examined our interview transcripts to identify statements or actions that reflected descriptions of resilience. We used PowerPoint and the digital collaboration platform Miro, to present and discuss findings in smaller groups together with colleagues, co-authors, and supervisors. Then, findings were presented to colleagues at different internal university workshops and subsequently at conferences. This concurs with Klein and Myers’ [38] principle of multiple interpretations and it adheres to Walsham’s [24] description of a ‘creative leap’; by filtering our emergent findings through the minds of others, a ‘creative leap’ resulted in the theorisation of resilience.

Interviewees representing different voices in various organizations and iterative processes of data collection and analysis, guided the process of deciding when we had reached a point of saturation, hence when there was lack of progression in generating themes. The data from the different sources were closely analyzed to identify concepts which then contributed to identify broader thematic areas for each country using an inductive analysis. These thematic areas were aggregated further to identify broader dimensions which will be discussed in the discussions chapter comparing and contrasting them across the countries.

### Role of authors

The Sri Lankan author was engaged in stakeholder discussions as well as implementation of the information system in addition to his role in contributing to data collection, analysis and articulation of the case study. One of the authors from Norway has contributed to the long-term capacity building activities in Sri Lanka in addition to his role in contributing to analysis and formulation of the case study. The author from Rwanda has been instrumental in the implementation of the digital technologies during the COVID pandemic and his lived experience contributed to the case study in addition to the interview and observations. Two authors from Norway were involved with data collection and analysis, formulating the case study section of this paper. They have both had a relatively neutral role, as outside observers [24] in the case study of Norway. An advantage of being an outside observer is that interviewees may have found it easier to respond frankly to questions [39], as none of the authors have had any personal investment in the project.

### Case studies

#### Sri Lanka

##### Background

Originating in China in December 2019, COVID-19 rapidly spread across Southeast Asia by early 2020. A surge in Chinese tourists threatened Sri Lanka's tourism sector. Despite lacking the advanced electronic infrastructure common in developed countries, essential for expansive surveillance, Sri Lanka swiftly responded. Leveraging open-source solutions, existing capacities, and governance frameworks, the Ministry of Health, along with other stakeholders, established a digital surveillance system to monitor the pandemic.

##### Rapid development & implementation of digital solutions

In January 2020, the Ministry of Health convened a crucial meeting to discuss the technicalities of creating a digital surveillance system for pandemic monitoring. Given the limited knowledge at the time, the system's design was to be agile, allowing frequent updates post-deployment. Assessing both the country's current capabilities and training needs, the Ministry chose the DHIS2, a health management information platform, as the central platform. The country had prior experience with this platform, enabling them to gather both aggregate and specific health data for various uses. Significantly, medical officers in health informatics played a vital role in implementing this platform both locally and nationally.

In anticipation of COVID-19, a platform was developed to monitor international visitors at airports and other entry points. By the close of January 2020, just before the country's first confirmed case, this system was ready. The design and execution were spearheaded by health informaticians from the Ministry of Health and the ICT agency of Sri Lanka, collaborating with HISP Sri Lanka, a local entity of the global HISP network responsible for DHIS2 implementation. The platform's adaptability enabled the team to accommodate evolving health needs, including quarantine tracking, contact tracing, community surveillance, ICU bed monitoring, and lab data collection. Within four months, these features were operational. Furthermore, to supplement the platform's built-in capabilities, a hackathon was organized in partnership with the ICT agency. This led to the development of additional tools, including a contact mapping visualizer, an ICU bed tracker, and a mobile tower-based contact tracing tool.

One interviewee commented,

“… it takes weeks or even months to get approval and implement a system within the ministry usually. But during the early days of the pandemic, it was hard to believe how stakeholders outside the ministry and sometimes even outside the country could collaborate with ministry officials and get systems designed and implemented…”

This highlights the level of collaboration which was present during the pandemic which led to rapid deliverables in system implementation.

Despite reduced disease prevalence in Q3 2020, efforts to customize and deploy systems persisted. Anticipating a second wave in Q4, the country bolstered lab monitoring and integrated community testing. The team successfully linked the COVID-19 lab system with the DHIS2 platform, forming a comprehensive digital surveillance ecosystem. In 2021, an immunisation module was introduced. It was designed to pre-register all Sri Lankan adults (over 16 million) for vaccination. This module was fully operational by the commencement of Sri Lanka's COVID vaccination drive. Mid-2021 saw an addition to the module: a feature for creating digitally verifiable vaccination certificates. Collaborating with the ICT Agency, the team integrated the DIVOC platform, designated for certificate generation, with the DHIS2 immunisation system.

A systems implementer highlighted the level of collaboration and how it produced a better experience to the clients of the health system.

"… this was the first time we were able to develop extensions on top of dhis2 and to connect it to another digital public good to produce an entire information system with a seamless integration between systems to provide a better experience to the clients. So it was not just a collaboration of stakeholders, but a collaboration of systems…“

To monitor the vaccination programme and deliver digital immunisation certificates to citizens for travel, the integrated solution provides a powerful digital system for capturing and visualising COVID vaccination information.

##### Leveraging on local capacity & stakeholder engagement

Using customized DHIS2 components and integrated solutions, the COVID-19 surveillance system emerged as a robust multisectoral information network. Led by the president, the national steering committee for COVID-19 exemplified strong collaboration among health, governmental, corporate, and international entities. Driven by a vision for long-term capacity-building in health informatics, the system notably offered real-time dashboards tailored for decision-makers, enhancing their decision-making capabilities.

The Sri Lankan approach was bolstered by international collaboration. The swift evolution of their system was facilitated through regular interactions with the open-source DHIS2 community and experts from the University of Oslo. Insights and metadata from Sri Lanka were further refined at the University and then released as a generic metadata package. This package has aided over fifty countries in establishing DHIS2-based COVID-19 surveillance. Feedback from the DHIS2 community enabled the global adaptation of tools like the contact mapping visualization app. Historically, IT staff training was centered at the district level, but COVID-19 restrictions impeded onsite training programs.

An information systems implementer stated,’

“…we had never conducted entire training programs remotely specially when collecting individual patient information. But the requirements around the pandemic was such that this was the best possible strategy to conduct training programs rapidly while not risking spread of disease aming health staff. So many parties contributed and the enthusiasm among health staff was the key to the success…”

To assist with training and support, video conferencing platforms and instant messaging groups were rapidly integrated. Healthcare professionals quickly adapted to and accepted this technology. While providing ICT infrastructure in various facilities posed challenges, the core team adeptly managed integration with existing and new information systems. One significant hurdle was navigating administrative structures and prioritizing implementation requirements.

In general, all the technological advancements around the information system made the decision-making process more efficient at all levels in the health hierarchy. An administrator stated,

“..the important thing about developing and implementing digital technologies was that we made it possible for health staff at all levels to track the progress whether positive or negative.. and to take decisions backed by data in a streamlined manner rather than haphazardly doing it using excel sheets.. this made the system stronger, the entire health system….”

**Fig. 1:**
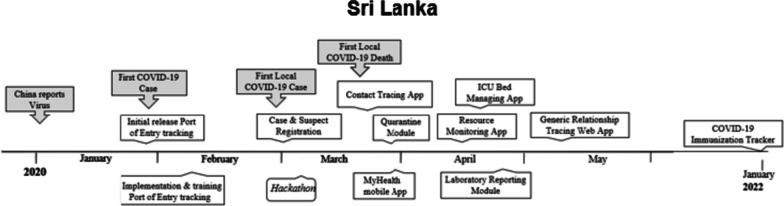
Timeline of events: Sri Lanka

Timeline of events of importance for the case study are depicted in Fig. 1.

### Rwanda

Rwanda is a small country in Central Africa with a population of 13 millions. While being categorised as a Least Developed Country by UN, Rwanda is among the fastest growing economies in the world and the government has a particular focus on digital transformation in their development agenda, which is important in our case. When the COVID-19 reached Rwanda, there were no available digital solutions that could be used to respond to the pandemic by tracing cases and contacts, link with laboratories, facilitate cross-border travels and to provide real-time statistics to help decision making.

As in the case of Sri Lanka, Rwanda was already using the DHIS2 platform for their general HIS and had good capacity in developing various IT solution using the platform. Based on templates for COVID-19 data standards distributed through WHO, and learning from the use of DHIS2 in Sri Lanka, Rwanda was able to rapidly build an integrated set of digital solutions supporting their pandemic responses. A task force with high level government support was leading the digital pandemic response and the national digital strategy combined with good IT infrastructure and Internet provided an important foundation.

The speed with which digital solutions had been implemented surprised the participants:

"In times of crisis, I was unaware that a system could be tailored and implemented within a week. Nevertheless, open-source solutions' potential to support during emergencies has been showcased."

One administrator highlighted,

“The presence of a task force has minimized the delayed approval procedures, resulting in accelerated implementation.”

By expanding the existing DHIS2 platform, Rwanda was able to rapidly respond to the need for monitoring of cases and contacts without having to develop new tools. As one member of the task force explained:

"Utilizing an existing platform and scaling it for capturing COVID cases proved advantageous for the Government, avoiding additional expenses due to pre-existing in-country capabilities."

The collaboration between multiple arms of government and the private sector, as well as other national institutions such as the National Identity Agency, has facilitated the deployment of digital solutions for COVID-19, by enabling the health sector to leverage existing data systems. Creation of the National command post played a big role in coordination of COVID-19 surveillance.

Rwanda’s digital solutions for COVID-19 emphasize patient access, for example, by enabling individuals to directly receive or track their own test results and to get their vaccination and COVID-19 certificates online.

Through integration of digital solutions and data from laboratories, testing, vaccination, border control, airport, etc., the entire testing process is now paperless, and users get their test results by sms and certificates online. Tracing and monitoring of cases and contacts using digital tools has reduced the burden on health workers and thus allowed the country to focus their limited capacity on delivering services to the most at-risk individuals, while maintaining consistent monitoring of other patients.

Management of all pandemic data from public and private sector into one integrated system enhances both understanding of the pandemic and the ability to coordinate and monitor the response interventions. It also simplifies data management, as all patients, regardless of where they are tested, receive their lab tracking number and results via the same DHIS2 platform.

Rwanda decided to leverage existing digital tools and capacity and to bring together expertise from different sectors. During the height of the pandemic, IT staff were pulled from their respective institutions to work together and support the testing and vaccination services.

**Fig. 2:**
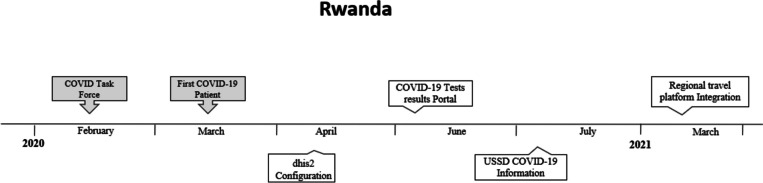
Timeline of events in Rwanda

Timeline of deployment of digital technologies during pandemic in Rwanda is highlighted in Fig. 2.

#### Norway

##### Background

This case study draws on data collection and data analysis from six municipalities in Norway who implemented the contact tracing system called Fiks contact tracing.

In Norway the health care sector has perfected routines and practices for contact tracing for almost 200 years. It is the municipalities´ responsibility to conduct contact tracing of their citizens infected by a communicable disease. The Infection Control Act [40] instructs municipalities in *what* their responsibilities are, such as implementing systems, routines and practices for infection control. However, as the Norwegian municipalities are autonomous, each municipality separately decides *how* to conduct work related to contact tracing. Due to a high vaccination rate against communicable diseases, municipalities have been able to handle minor outbreaks of infections with phone, pen and paper and spreadsheets. For instance, in a population close to 6 millions, there are between 150 and 200 registered cases of Tuberculosis per year [41]. Click or tap here to enter text..

As a NIPH senior advisor said:

*“We have not experienced contact tracing at this scale before. When there is an outbreak we have routines for surveillance, outbreak investigation, and we have interviewed the infected persons. Today it is a totally different form of contact tracing than before. With measles, we map our the close contacts, but most of the close contats are immunised against measles. The scale of the work is therefore much smaller. There has not been a need for such [digital] tools as [the ones] we have now. For ebola the same contact tracing [as with COVID-19] is relevant but that has not been necessary in western countries.”* (NIPH senior advisor).

COVID-19, being a novel infection with no vaccination, had the ability to spread everywhere. On 26 February, Norway registered its first case of COVID-19. Seeing how quickly COVID-19 could spread, traditional contact tracing tools did not suffice. Health officers in the municipality where the first case was identified, initiated a search for potential digital contact tracing systems (CTS) that could help them with contact tracing.

##### Rapid development & implementation of digital solutions

Although the Norwegian municipalities are autonomous, they receive different forms of support from the Norwegian Association of Local and Regional Authorities, also known as the municipal sector organization (KS). This includes information technology (IT) support through a software-as-a-service platform called Fiks. KS became an invaluable contributor to the digitalization process.

Based on a recommendation from the World Health Organization (WHO) and Centre for Disease Control and Prevention (CDC), an implementation of DHIS2 was initiated. DHIS2 is a customizable open-source system with Application Programming Interfaces (APIs), with a COVID-19 package, developed by Sri Lanka. The system could be adapted to local workflows with low implementation costs. Finally, HISP Norway had available implementation competence.

As a chief municipal physician said,

*“I contacted DHIS2 [HISP Oslo] to try and build up something in Norway. To document and report COVID-19. All [health] registers in Norway are top-down, while this is a bottom-up [health register] from the municipalities. That it was an open-source tool was very important.”* (Municipal chief physician).

Together with KS and HISP Oslo, municipal health officers initiated a bottom-up digitalization process. As the municipalities´ IT service provider, KS implemented the digital CTS, called Fiks contact tracing, on KS digital platform called Fiks. A first pilot was ready in May 2020. KS led a democratic digitalization process, creating a user forum consisting of regular meetings where representatives from engaged municipalities report errors, describe desired new functionality, and share user experiences with KS and with other municipalities.

A KS administrator explained it like this,

*“The resources [participants] in the user council were very active, from both large and small municipalities. In [some] periods we had weekly meetings with the municipalities. It was important to sit close to them. [Together] we lifted tasks up and down. For some municipalities [a task] was not important but it was still relevant when we deployed it. The user needs are governed by the municipalities. KS governs the administration. We involved the municipalities in testing and acceptance testing.”* (KS administrator).

Contrary to other digitalization processes within the healthcare sector, the digitalization of COVID-19 has been labelled the speediest digitalization collaboration in Norwegian history [42]. According to KS´ project manager, all contributors were characterized by a unique desire to help, in Norwegian labelled as “dugnad”.

##### Leveraging on local capacity & stakeholder engagement

Initially, there were challenges related to Fiks contact tracing being a stand-alone (SILO) system with no integrations towards other HIS. Contact tracers could spend up to 2 overtime hours per day because they had to register information about positive COVID-19 cases several times in different HIS. By utilizing open boundary resources in DHIS2, and with economic support from NIPH, KS developed integrations between Fiks contact tracing and the national population register (NPR), NIPH´s laboratory database for transfer of laboratory results, and NIPH´s clinical report database for automatic transfer of clinical reports from Fiks contact tracing.

**Fig. 3:**
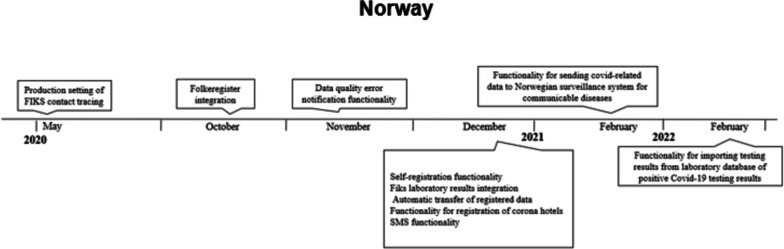
Timeline of events: Norway

Additionally, a self-registration module was made available for the public, as well as automatic transfer of a registered positive case from one municipality to another. Figure 3 below highlights the timeline of implementation of some important functionalities in Fiks contact tracing.

Municipalities in Norway are bureaucratic and hierarchical, and each unit is organized as a silo, where distinct disciplines work in different departments, thus not facilitating interdisciplinary work.

A municipal project manager described it like this:

*“We think differently, work differently, set us up in a different way, we think interdisciplinary and cross sectional.” (Municipal project manager).*

This was supported by another municipal project manager who said:

*The goal must be extremely clear when you work cross-sectional. [In the municipality] the focus is on prioritization, services and the like. What is important to me is not important to others. During the pandemic [this was different], what was important was important to all.* (Municipal project manager).

The COVID-19 pandemic necessitated speedy interdisciplinary collaborations. At the onset of the pandemic, a municipality project manager established an interdisciplinary team with members who had knowledge of for instance law, health, IT, project management and economics. The distance between the one asking a question and the one answering it was minimized.

The municipal project manager described it like this:

*“Some always worked from home because we were not supposed to be so many together the same time [due to chances of infection] so it was difficult to get an overview of all the stakeholders. Then we got a contact person in the IT department and a developer [only for us] from the start. From then on someone had the overview of what was important. They got a strong feeling of ownership to something and attended our meetings. At the most we were twelve people in my team. We had standups every day on zoom, at least for half an hour, and two longer meetings during the week. The team was represented [by representatives] across the municipality: digitalization, ICT operation, analysis, health, and welfare technology. People could come to us, we became the hub in the middle.”* (Municipal project manager).

For instance, a question related to law, if something was allowed to develop or not, could be answered within the team. The interdisciplinary team´s members from the IT-department created integrations between Fiks contact tracing and the preparedness team, which enabled one-click-production of reports. Thus, immediate access to the different subject expertises helped facilitate a rapid digitalization.

## Discussion

In this chapter, we analyze and discuss the case studies to identify the resilience attributes emerged through the case studies and to derive the socio-technical determinants of resilience through inductive data analysis.

A resilient HIS can adapt to continuous change without changing its identity [8, 10]. An HIS is resilient when it is able to evolve and adapt continuously while staying within crucial thresholds (Ibid.). Resilience is about persistence, adaptability and transformability (Ibid).

Several attributes of resilience are highlighted in the literature. However, we identify a few crucial properties of resilience from the empirical data we draw from the study across the three countries.

### Attributes of IS resilience

#### Bouncing back & bouncing forward

Drawing on Folke et al.´s [8] description of resilience, we argue that an IS is resilient when it adapts to a crisis by bouncing forward, which may result in a substantial change in use of technology, practices and routines. Similar notions have been described in recent IS papers, like Heeks and Ospina [18] who focus on adaptation and transformation to describe resilience of an IS, and Amir and Kant [43] who link socio technical resilience to transformability. We continue in this stream of understanding resilience as bounce-back and bounce-forward.

The transformation may take place through several adaptive steps. For the Norwegian case, we describe four of the main steps municipalities bounced forward. The first step entailed municipalities establishing corona clinics and manual CTS. Due to a rapid increase in infection rates, the manual CTS did not suffice. Thus, a second step forward entailed municipalities adapting by implementing the digital CTS Fiks contact tracing, and the employment of contact tracers who could register data about infected individuals. Although Fiks contact tracing made it easier to keep an overview of infected individuals and their close contacts, it was initially implemented as a SILO system. Thus, contact tracers spent much overtime hours to manually create reports on aggregated contact tracing data. A third step forward therefore entailed developing different integrations towards other IS within the municipality as well as national IS and HIS. With the COVID-19 Omicron variant, the infection rate rose to new record levels. The municipalities did not have enough resources to register all infected individuals and their close contacts. A fourth step forward thus called for the deployment of a self-registration module, which made it possible for citizens to register positive self-tests. That way, the municipalities were able to keep an overview of infected citizens without experiencing a high number of burned-out employees.

Bouncing back is explained as the returning back to what was before the crisis or the shock [18]. Norwegian municipalities have not completely bounced back after the COVID-19 pandemic. They have established new routines, practices, technologies, and knowledge systems for contact tracing. Contact tracing teams have been co-located with vaccination teams. While one municipality has been continuously working to create a preparedness team during the COVID-19 pandemic that will be ready to handle a new health crisis, some municipalities have just started this work, and yet others have almost bounced back. Fiks contact tracing is, however, in continuous development and currently it has available functionality for contact tracing of infectious diseases such as tuberculosis, monkeypox, meningitis, MRSA and E.coli. Several Norwegian municipalities would like to use Fiks contact tracing as a platform for routine surveillance and embed it to existing surveillance workflows suggesting an approach to combat disease outbreaks in future. We argue this as a bounce-forward aspect of the health system driven by digital technology.

In the contexts of Sri Lanka and Rwanda, the initial requirement of the countries was to develop a digital system to conduct disease surveillance for COVID-19 in times of crisis to effectively function health systems as during the pre-pandemic era. However, both countries realized the potential of an integrated digital information system emerged during the pandemic to conduct routine surveillance, mass vaccination campaigns and management of commodities such as ICU beds. Thus, opportunities were created in both the countries to utilize digital systems in an integrated environment linking the existing systems as well as to innovate components in the existing environment as opposed to building new technologies leading to fragmentations. In the context of Sri Lanka plans were underway to develop a routine digital health management information system based on learnings around use of the platform during the pandemic. Such measures provide empirical basis to define the bounce-forward nature of the response of the health system to transition to a status further to the where it existed before the shock of the pandemic.

#### Agility

According to researchers, modern firms must be agile in order to prosper [44]. An organization must find a way to balance the demands of its external environment with the speed with which it can reorganize its internal resources in response if it wants to be considered agile. In response to the demands of a volatile, rapidly changing environment, research has looked at how companies might dynamically restructure their resource base to achieve new capabilities. The degree and speed of this reconfiguration that an organization is capable of closely relates to the idea of its agility [40]. The term "organizational agility" (OA) describes a company's capacity to recognise external changes and react to them quickly and effectively [45]. In this paper, we try to focus on a broader country and municipality level than what is available in the literature, which is mostly focused on the organizational level.

Drawing on the three case studies, it was evident that there was increased acceptability for change in the health sector during the times of the pandemic. All three case studies reveal to some extent the general inertia of decision-making and long approval process involved in design and implementation of information systems prior to the pandemic. During the pandemic there was increased presence of stakeholder engagement within the Sri Lankan and Rwandan ministries as well outside expediting the entire process. This is a crucial element of agility and responsiveness which contributed to the rapid development of components of the information system. In addition, the ministries were sensitive and receptive to the changing requirements arising from the changing disease trajectory as well as feedback received from users. This is vital in establishing the responsiveness element. In the context of Norway, the interdisciplinary teams that were established by actors involved in the digitalization of contact tracing, played an invaluable role. They were responsive to the requirements that arose with the pandemic and rapidly and responded to the user and domain requirements.

Thus, we believe both the rapidity and responsiveness are crucial components of agility which in turn contributed to the resilience of the information system of the three countries during the pandemic.

### Socio-technical determinants of process of building resilience

Socio-technical resilience has to do with transformability, and it entails a socio-technical system being able to change from one configuration to another due to a disruption or a shock [43]. This includes the ability to scale up and down the number of users and the digital system according to new rules and restrictions that influence contact tracing and COVID surveillance practices, due to changes in epidemiology. For instance, increase and decrease in the infection rates due to new variants of virus influenced days in quarantine and isolation, which had to be represented in the digital CTS as well as in the contact tracers´ learning- and information material. In this chapter, we identify major determinants in the process of building resilience in information systems based on the findings from the case study.

#### Existing local capacity

Introducing multiple solutions to a country that has a lack of capacity could lead to serious issues related to sustainability, based on experience from the Ebola outbreak [13]. There are several different types of knowledge and skills enhancement in the domain of IS. The ability to govern the direction, implementation, and coordination of IS through the creation of standard operating procedures (SOPs) and high-level coordinating entities like working groups or task forces are even more crucial [46].

The contact tracing teams in Norway have consisted of highly interdisciplinary teams, with team members being doctors, nurses, physiotherapists, pilots, flight attendants, police investigators, and shopkeepers, to name some. Their different backgrounds and experiences have influenced their view on contact tracing routines and practices, including systems´ functionalities. Contact tracers with little or no experience from the health care sector have often questioned comments like “it has always been done like this”, or “it is not possible to digitalize this process” and suggested other solutions or made changes themselves. This corresponds with how Walker and Salt [10] describe resilient people with a positive capacity being more solution-oriented than problem-oriented.

In the context of Sri Lanka and Rwanda, both countries had long-standing experience on building digital information systems in the healthcare sector on the free and open-source platform, DHIS2. The drive for using open-source solutions may partly be due to the low availability of financial resources to procure and maintain proprietary solutions. However, it is understood from the 2 case studies that both countries possessed experience in using the dhis2 platform for a variety of use cases and local capacity was rooted at all levels in the health hierarchy in design, customization and use of the platform for routine health data requirements. Thus, during the early phase of COVID-19 when global travel was limited due to restrictions, both countries were still able to not only design but also establish a digital system for data requirements around COVID-19 by utilizing local resources. While COVID-19 was a shock and draining too much resources from the health system, the countries were still able to devise a digital system and implement it across the country due to the existing local resources.

#### Networking & collaboration

The rapid bottom-up innovation in Norwegian municipalities necessitated an interdisciplinary collaboration across the different units in the municipalities. Hierarchies were flattened and traditional bureaucratic processes were set aside. The digital CTS with its integrations facilitated new communication and collaboration processes both within and across municipalities, for instance enabling digital transfer of data. This shows how HIS may create new path dependencies as well as facilitate multi-scalar effects. It corresponds with Folke et al´s [8] view on resilience as a potential for transformability, and Floetgen et al.’ [23] argument about resilience being built transformatively.

In the LMIC context, both Sri Lanka and Rwanda demonstrate collaboration within ministries as well as with external entities as a major factor contributing to rapid implementation of digital solutions in the health sector. The peculiar factor to notice is the level of collaboration observed during the pandemic under stressful conditions was much greater than what was generally observed in routine service delivery. The case studies testify that the collaborations were crucial for the implementations during the pandemic amidst stretched resources. Other than in-country collaborations, the open-source network around the dhis2 at regional and global level facilitated sharing of expertise across the three countries involved in the study. The learnings of collaborations and sharing from LMIC context supported the implementation in Norway.

#### Technology/platform

One major similarity observed in the 3 countries in the study is the core platform around which the digital technologies for COVID-19 was built on. Hsu et al. [47] argue from a quantitative survey that openness of technology and support from top level managers catalyses adoption and innovation. While the free and open-source nature was of the DHIS2 platform was a determinant for the historical adoption of the platform in resource-limited Sri Lanka and Rwanda, it is interesting to observe how this property of the platform contributed to its adoption in Norway during the time of the pandemic.

Flexibility is a key component observed in the DHIS2 platform which contributed its use in all three countries when the disease trajectory of the COVID-19 was changing. Ability of developing web applications and creating integrations was a salient requirement in Sri Lanka and Rwanda during mid 2020 following the implementation of the solution in early phase of the pandemic. The platform essentially was resilient to accommodate such requirements as well as to scale it to manage the entire population data during the COVID vaccination. On the other hand, the ability to create integrations and opensource nature allowing further development on the codebase was a core requirement for its deployment in Norway, a country which has a complex digital ecosystem with integrated components. Based on the case studies it is evident that the access to the codebase and the flexibility of the platform cleared the path to more stakeholders adopting and contributing to the implementation of the digital technology on the DHIS2 platform.

#### Enabling antecedent conditions

Sri Lanka had invested in long term capacity building on health informatics and placing the skilled graduates at all levels in the health hierarchy for over a decade. Rwanda had a vision for implementation of digital technologies in the health sector, adopted a flexible open-source platform and developed capacity on using and maintaining the platform. In the context of Norway, the country already possessed sufficient resources and advanced integrated digital technologies. In addition, Norway was the central hub for design and coordination of the global digital platform which was the common solution implemented across the three countries. All the above conditions were pre-existing in the context in which the digital technologies were implemented during the pandemic. While the local capacity, collaborations and properties of digital platforms contributed to the resilience observed during the pandemic, a critical analysis could identify the necessity of enabling antecedent conditions mentioned above in achieving the resilience. In other words, we argue that achieving resilience in information management using digital technologies would be challenging if enabling antecedent conditions are not existing in a context.

Once we identified the major determinants of the process of building resilient information systems, we focus on relating how it contributes to the broader health system based on the case studies. We identified mainly through the interviews in Sri Lanka how a digital information system impacted the health system processes. The healthcare administrators highlighted that availability of information enabled them to arrive at decisions based on available data rather than speculating. In addition, the digital system expanded the evidence-based decision-making to all levels in the health system hierarchy and not just the national level. This was a significant contribution for management of the health system in general and thereby contributing to making the resilience of the broader health system. However, in the case of Norway the information system supported the decision making primarily at health facility level. However, there were challenges in utilizing the initial contact tracing system at national and subnational level due to constraints related to integrations, existing privacy and standardization practices in the health sector. This highlights the long process of maturity required in the context of a more developed country which has existing standards and governance mechanisms in use and management of data.

There are several limitations in the study. We try to portray determinants of resilient information systems based on our empirical data in the context of COVID-19 pandemic. The study does not explore the context of routine information systems other than several inferences in the case studies. We also bring about determinants based on context of 3 countries. However, we attempted to ensure the representation by including two countries in low and middle income setting and one high income country. In the data collection process, we opted to study national system based on access and convenience. This may have led to omission of few other digital systems which were functioning in the country (eg: omission of Remin system of Norway which was also used for contact tracing). Since the study was conducted during the height of the pandemic, the interviews were mostly conducted online in some countries. Due to this, certain important steps in the process of digitalisation may not have been captured.

## Conclusion

This paper delves into the resilience of health systems, specifically focusing on HIS, a key component among the World Health Organization's six health system pillars. Our findings emphasize the socio-technical factors influencing resilient systems: local capacity, collaboration, adaptable digital platforms, and favorable antecedent conditions. True resilience is achieved when systems are agile, continuously evolving to address dynamic needs.

Drawing from empirical case studies, practical implications in the health sector emerge. A notable suggestion is refining policy-making processes, necessitating the restructure of both the public sector and its interaction with the private realm. Effective governance embraces all these foundational elements, emphasizing cross-sectoral and interdisciplinary collaboration. Therefore, policy-making, budget allocations, and strategic planning must champion such collaborations.

The study outlines key factors nations should consider for crafting resilient information systems, which subsequently fortify overall health systems. Future research should probe deeper into these determinants, offering insights into establishing resilience within specific national contexts. A paradigm shift in planning and budgeting is imperative. Instead of individual unit allocations, subnational levels must adopt a holistic, citizen-centric approach, fostering collaboration across units to enhance services for their constituents.

## Data Availability

The datasets generated and/or analysed during the current study are not publicly available since not being permitted to share interviews due to privacy concerns highlighted in ethics approval. However, analysed categorised data could be shared upon request. Please contact corresponding author, Pamod Amarakoon (pamodm@gmail.com) for any clarification and data requests.
